# Does the extent of collaterals influence the severity of the myocardial injury as assessed by elevation in biomarkers?

**DOI:** 10.34172/jcvtr.2021.08

**Published:** 2021-01-28

**Authors:** Gajendra Dubey, Kamal Sharma, Iva Patel, Zeeshan Mansuri, Vishal Sharma

**Affiliations:** ^1^Department of Cardiology, U.N.Mehta Institute of Cardiology and Research Centre (UNMICRC), Civil Hospital Campus, Asarwa, Ahmedabad-380016, Gujarat, India; ^2^Research Department U.N.Mehta Institute of Cardiology and Research Centre (UNMICRC), Civil Hospital Campus, Asarwa, Ahmedabad-380016, Gujarat, India

**Keywords:** Cardiac Coronary Collaterals, Troponin I, CPK-MB, Biomarkers

## Abstract

***Introduction:*** Quantitative analysis of cardiac biomarkers, troponin I and CPK-MB, estimates the extent of myocardial injury while extent of benefit from coronary collateral circulation (CCC) to protect myocardium during acute myocardial infarction (AMI) needs validation. We analysed if the extent of collaterals had impact on baseline biomarkers at the time of coronary angiogram.

***Methods:*** We analysed 3616 consecutive patients who presented with AMI and underwent invasive coronary angiography (CAG) with intent to revascularisation with biomarkers assessment at the time of CAG. CCC to Infarct related artery (IRA) were graded as per Rentrop grading viz. poorly-developed CCC (Grade 0/1 as Group A) and well-developed CCC (Grade 2/3 as Group B).

***Results:*** Both groups (A and B) were matched for demographics, traditional risk factors, SYNTAX 1 Score, time to CAG from onset of angina and eGFR. 36.59% of patients had Non-ST segment elevation myocardial infarction (NSTEMI) as compared to 63.41% ST -segment elevation infarction (STEMI). Overall Troponin I (*P* =0.01, *P* =0.01) and CPK MB (*P* =0.00, *P* =0.002) values were lower in group B in both NSTEMI and STEMI groups respectively. Troponin I and CPK-MB were significantly lower in group B [with NSTEMI for SVD (Single vessel disease) (*P* =0.05) and DVD (Double vessel disease) (*P* =0.04),but not for TVD (Triple vessel disease) and with STEMI in SVD (*P* =0.01), DVD (*P* =0.01) and TVD (*P* =0.001)].

***Conclusion:*** Patients with well-developed coronary collaterals had a lower rise in biomarkers in AMI as compared to those with poor collaterals amongst both NSTEMI and STEMI groups.

## Introduction

Measurement of serum Troponin I and CPK-MB is considered as a standard test in the work up of acute coronary syndrome. Their Quantitative assessment plays a pivotal role in the diagnosis, risk stratification and management of coronary artery disease. Patients with well-developed Coronary collateral circulation (CCC) may have an alternate source of blood supply to the occluded vessel territory in AMI. CCC may hence reduce the extent of myocardial injury in AMI due to flow-limiting stenosis or occlusion.^1^ The late recruitment of pre-formed CCC has been shown to result in better recovery with late reperfusion of myocardium of the infarct related artery.^2^ Though the development of collaterals is usually an adaptive mechanism to chronic myocardial ischemia serving as alternate source of blood flow to significantly obstructed coronary vessels territory, recruitment of pre-existing collaterals in acute settings may also be of benefit in myocardial preservation in face of acute ischemia.

However, in acute coronary occlusion, in absence of substantial CCC, myocyte cell death in the subtended area of the occluded artery that starts at approximately 60 minutes tends to complete within a few hours, with biomarkers rising in proportion to the injury.^3^ On the other hand, in occluded vessels with well-developed collaterals, the area of jeopardized myocardium may receive some blood flow, and this may prevent irreversible myonecrosis.^4^

There is paucity of data about AMI patients with grading of CCC and their impact on extent of rise of these biomarkers especially amongst Asian Indians ethnicity. In patients with well-developed CCC, due to alternate source of blood supply, the biomarkers may have lower values for extent of myocardial injury. This may hence reflect the protective effect of the collateral circulation against the myocardial injury. The present study was designed to evaluate the impact of extent of CCC as evident on angiography on the extent of elevation of CPK MB and Troponin I (biomarkers value) at the time of angiogram in myocardial infarction (NSTEMI and STEMI) patients.

## Materials and Methods

### Study design

**Figure 1:**
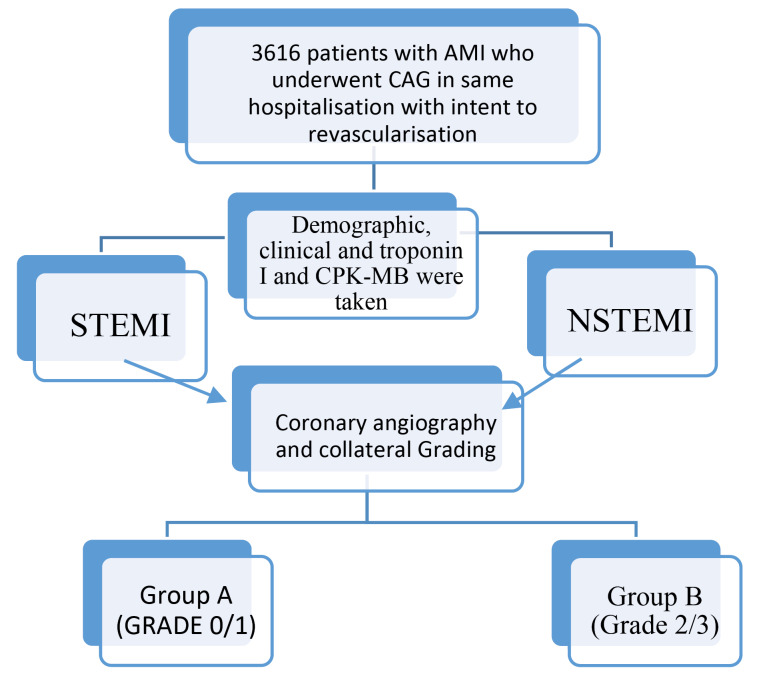
Central illustration- FLOWCHART

This study has a retrospective, observational, all comers, and consecutive cohort design. The flowchart of the study was shown in Figure 1.

### Clinical data collection:

Between January 2017 to June 2017, complete medical records of all consecutive patients (n= 3616) who were diagnosed with myocardial infarction (NSTEMI and STEMI) and had undergone coronary angiography (CAG) during same hospitalization with intent to revascularisation as per the prevalent ESC guidelines and their details were collected from medical records department.^5^ Variables like demographics, clinical and angiographic variables including Rentrop’s grade of coronary collaterals to infarct related artery (IRA) and SYNTAX I score were collected. Patients were excluded if they had history of acute coronary syndrome other than current episode within last 3 months or has ever undergone coronary revascularisation, were in NYHA class IV heart failure, had associated severe valvular heart disease, presence of co-existent inflammatory disease (e.g., rheumatoid arthritis), acute or chronic renal failure, severe hepatic diseases, or pregnancy. Patients with myocardial infarction who didn’t undergo coronary angiography during index hospitalization were also excluded from the study. The study was approved by the institutional ethics committee.

### Grading of coronary collaterals:

Collateral vessels were graded according to the Rentrop classification: grade 0 = no filling of collateral vessels; grade 1 = filling of collateral vessels without any epicardial filling of the occluded; grade 2 = partial epicardial filling of the occluded artery; grade 3 = complete epicardial filling of the occluded artery.^6^ Based on angiographic data, patients were grouped into two categories; group A comprised of those with Grade 0 and I collateral and group B with Grade II and III collaterals.

### Blood samples and analyses:

Values of assays from blood samples collected at the time of angiogram were used for the study to derive its correlation with angiographically visualised CCC. Cardiac troponin I measurements were done using “ARCHITECH STAT high sensitive Troponin-I assay” and CK MB measurements were done using “MULTIGENT CK MB assay”. Both samples were analysed using counter ARCHITECT PLUS (ci 4100) (Abbott). Normal reference levels, as recommended by manufacturers, were 0.010 to 0.05 ng/ml for Troponin I and 30-170 U/L for CK-MB.

### Statistical analysis

The data analysis was done using “IBM SPSS version 25” (IBM Corp., New York, USA). All collected data was set on Microsoft excel data sheet and variables were entered into a common database for analysis. Quantitative variables are expressed as mean ± standard deviation, and qualitative variables are expressed as percentage. Comparison of parametric values between groups was performed using the independent sample *t*-test and multivariate analysis. Categorical variables were compared using the Chi-square test. A nominal significance was taken as a two-tailed *P*< 0.05.

## Results

Table 1 represents the baseline characteristic of population. All the traditional risk factors were distributed evenly between both group A and B. Patients in group B ( grade 2 and 3 ) were older and prevalence of male gender was higher in group B than group A. Both the groups had higher prevalence of males as compared to females. The traditional risk factors namely Diabetes mellitus, hypertension, consumption of tobacco, family history of premature CAD, baseline eGFR, SYNTAX 1 Score on angiogram and LVEF on Trans-Thoracic Echocardiography (TTE) were evenly matched and are mentioned in Table 1 for comparison. Troponin I and CPK-MB were significantly lower in group B (with well-developed CCC).

**Table 1:** Baseline characteristics of the population in both Groups

**Variables**	**Group -A** ** N=2949(81.55%)**	**Group -B** ** N=667(18.45%)**	***P*** ** value**
Age	57.40±10.50	58.38±9.56	0.03*
Male	2352 (79.8)	555 (83.2%)	0.05
Female	597 (20.2%)	112 (16.8%)	
HTN	606 (20.5%)	134 (20.1%)	0.83
DM	509 (17.3%)	118 (17.7%)	0.83
Tobacco	413 (14%)	91 (13.64%)	0.86
Dyslipidemia	489 (14%)	103 (15.44%)	0.51
Family history	314 (10.65%)	76 (11.39%)	0.62
Duration of angina since onset to presentation (minutes)	690 + 178	701+ 203	0.16
Mean LVEF (%)	42.07±9.64	41.32±10.44	0.07
Mean Syntax I score	18.5 + 12.5	19.12±3.2	0.20
Mean eGFR	44.05+13.07	43.01+12.5	0.07
Mean Troponin I	4.83±12.61	2.25±5.49	0.00*
Mean CPK-MB	35.43±77.09	23.57±42.66	0.00*
LAD- IRA	1736 (58,8%)	399 (59.8%)	0.68
LCX- IRA	589 (19.97%)	127(19%)	0.62
RCA- IRA	624(21.15%)	141(21.13%)	0.98

Abbreviations: HTN, hypertension; DM, diabetes mellitus; LVEF, left ventricular ejection fraction; eGFR, estimated glomerular filteration rate ; LAD, left anterior descending; IRA, infarct related artery; RCA, right coronary artery, LCX; left circumflex *Statistically significant

Table 2 shows the comparison of biomarkers between collateral grade (1,2) and collateral grade (2/3) in AMI patients. The value of Troponin I in both STEMI and NSTEMI was significantly (*P* = 0.01, *P* = 0.000) lower in those with good collateral (grade 2/3). Similarly, the value of CPK MB was significantly lower in group B with good CCC grade (2/3) in both STEMI (*P* = 0.01) and NSTEMI (=0.002).

**Table 2:** Comparison of biomarkers value in STEMI and NSTEMI in different grades of collaterals

	**Biomarkers**	**Group -A** **N=2949 (81.55%)**	**Group -B** ** N=667 (18.45%)**	***P*** ** value**
NSTEMI	Troponin I	5.61±13.26	3.34±7.09	0.01*
	CPK-MB	42.83±79.63	30.28±49.65	0.01*
STEMI	Troponin I	4.41±12.21	1.52±3.93	0.000*
	CPK-MB	31.13±75.35	19.05±36.63	0.002*

Abbreviations: NSTEMI, non-ST elevation myocardial infarction; STEMI, ST elevation myocardial infarction *Statistically significant

Comparison of Troponin I values between Group A (grade 0/1) and Group B (Grade 2/3) according to number of vessels with critical stenosis with respect to STEMI and NSTEMI is presented in Table 3. In NSTEMI patients, the value of troponin I was significantly lower in Group B (grade 2/3) in single vessel disease (SVD) (*P* = 0.05) and double vessel disease (DVD) (*P* = 0.04). In Triple vessel disease (TVD), though the value was lower in Group B (grade 2, 3 CCC) but it was statistically not significant. Amongst STEMI patients, the troponin I value was significantly lower in Group B (Grade 2/3 CCC) in SVD (*P* = 0.01), DVD (*P* = 0.01) and TVD (*P* = 0.001).

**Table 3:** Comparison of Troponin I value between Grade (0/1) and Grade (2/3) in STEMI and NSTEMI in relation to no. of vessels blocked

	**NSTEMI N=1323**		***P *** **value**	**STEMI N= 2293**		***P*** ** value**
	**Group -A** ** N=1055 (79.74%)**	**Group -B** ** N=268 (20.26%)**		**Group -A** ** N=1894 (82.60%)**	**Group -B** ** N=399 (17.40 %)**	
SVD	6.39±14.96	4.48±8.65	0.05	4.25±12.11	0.97±3.44	0.01*
DVD	6.73±14.35	3.10±6.09	0.04	4.86±12.91	32.37±77.19	0.01*
TVD	3.14±7.92	2.97±6.89	0.74	3.39±10.99	1.57±3.95	0.001*

Abbreviations: SVD, single vessel disease; DVD, double vessel disease; TVD, triple vessel disease *Statistically significant.

Table 4 represents the comparison of CPK MB according to number of vessels with critical lesion on angiography. Amongst NSTEMI patients, the value of CPK MB was significantly lower in Group B (grade 2/3 CCC) in DVD (*P* = 0.02) and TVD (*P* = 0.01). In STEMI patients the value was significantly lower in Group B (grade 2/3 CCC) collaterals in SVD (*P* = 0.04), DVD (*P* = 0.001) as well as TVD (*P* = 0.05).

**Table 4:** Comparison of CPK-MB value between Grade (0/1) and Grade (2/3) in STEMI and NSTEMI in relation to no. of vessels blocked

	**NSTEMI**		***P*** ** value**	**STEMI**		***P*** ** value**
	**Group -A** ** N=1055 (79.74%)**	**Group -B** ** N=268 (20.26%)**		**Group -A** ** N=-1894 (82.60%)**	**Group -B** ** N=399 (17.40%)**	
SVD	40.20±79.81	34.21±51.48	0.22	30.77±76.66	13.34±23.04	0.04*
DVD	44.56±77.36	33.24±51.61	**0.02**	32.37±77.19	19.78±32.98	0.001*
TVD	41.32±76.86	28.86±49.44	**0.01**	28.32±63.74	21.81±44.48	0.05*

Abbreviations: SVD, single vessel disease; DVD, double vessel disease; TVD; triple vessel disease *Statistically significant.

## Discussion

In this study, we investigated the impact of presence and extent of CCC to IRA on traditional cardiac biomarkers namely Troponin I and CPK-MB. CCC may potentially provide an alternative source of blood supply to the occluded coronary vessels’ myocardial territory in AMI patients. In our study, we found that presence of poor collateral circulation (CCC grade 0/1) was associated with significantly higher biomarker values as compared to presence of well developed collateral circulation (CCC grade 2/3). We also intended to analyse the effect of extent of CCC in subgroup of number of vessels blocked in STEMI and NSTEMI patients in our Asian Indian cohort. The extent of rise in the biomarkers is influenced by not only the number of vessels diseased and criticality of it but also by the site of lesion viz whether it is proximal or distal thus reflecting the extent of myocardium at jeopardy.

Our results are similar with other previously published studies in other populations. Older age and male gender have been found to be significantly associated with well-developed CCC.^7-11^ Prior studies have shown that cardiovascular risk factors such as hypertension, smoking, diabetes and dyslipidaemia were evenly distributed in both groups of collateral which is consistent with our study.^9,12^

Our results of acute cardio-protective effect of presence of well-developed CCC (grade 2/3) are consistent with previously published data.^9,13,14^ In fact, the biomarker value decreases as the collateral grade increases.^15,16,17^

Our study is unique for analysing biomarkers sample being drawn at the time of angiogram. The study has given new insights into the impact of well-developed collaterals on traditional biomarker values in AMI patients in Asian Indian population. It also gave us insight about number of vessels involved and type of AMI (STEMI vs NSTEMI). AUC (area under the curve) of the biomarkers done serially could be another and probably better way to analyse the impact of collaterals however same was not considered here as it could have been easily confounded by multiple factors like extent of revascularisation, TIMI /TMB grade post procedure, contrast nephropathy, baseline eGFR, age , overall duration of hospitalisation and outcomes. However, the same can be considered in future studies to consolidate the findings of this study.

The protective effective of well developed collateral circulation was seen in both STEMI and NSTEMI patients. Overall Troponin I (*P* = 0.01 and *P* = 0.00) and CPK MB (*P* = 0.01, *P* = 0.002) values were lower in group B in both NSTEMI and STEMI respectively. Troponin I was significantly lower in group B with NSTEMI for SVD (*P* = 0.05) and DVD (*P* = 0.04) but not for TVD. In STEMI, it was lower in group B in SVD (*P* = 0.01), DVD (*P* = 0.01) as well as TVD (*P* = 0.001). CPK-MB value was significantly lower in group B in NSTEMI with DVD (*P* = 0.02) and TVD (0.01) and in STEMI with SVD (*P* = 0.04), DVD (*P* = 0.001) as well as TVD *P* = (0.05).

A study by Lazoglu et al ^18^ showed that higher CPK-MB value was an independent predictor of poor collateral circulation in NSTEMI patients. Data regarding the impact of presence of collateral circulation on troponin levels is sparse. A prior study using Troponin T showed similar findings as our current study with higher levels in those with poor coronary collaterals.^15,17^ Studies by klopfer et al^19^ and Habib et al ^20^ were also on similar lines.

## Applied and translational implication

Higher grade of collaterals may limit the extent of myocardial injury both in STEMI and NSTEMI as assessed by values of troponin I and CPKMB at the time of coronary angiogram. Efforts to need to be reoriented towards development of therapies and lifestyle and rehabilitative therapies apart from genetic means towards development of therapeutics towards the same.

The “recall bias” of the time of onset of angina and late presentation from the onset to time of angiogram could have confounded our results despite no statistically significant difference between both the groups at baseline. Since we analysed the baseline biomarkers at the time of angiogram, AUC could be used in future studies to consolidate the findings of this study.

## Conclusion

Patients presenting with acute coronary syndromes and having well developed coronary collaterals to IRA show lower rise in traditional biomarker values in comparison to those with poorly developed coronary collaterals. However, higher grade of CCC did not have significantly lower value of Troponin I in TVD and CPKMB in SVD in NSTEMI.

## Acknowledgments

We would like to acknowledge all the hospital staff and Krutika Patel from research deparment for their support in conducting the present study.

## Competing interest

None.

## Ethical approval

The study was approved by institutional Ethical committee of U.N.Mehta institute of cardiology and research centre, Ahmedabad. (UNMICRC/ALLIED/2017/09)

## Funding

U. N. Mehta Institute of Cardiology and Research Centre (UNMICRC), Civil Hospital Campus, Asarwa, Ahmedabad-380016, Gujarat, India.
